# The development of the Quality Indicator for Rehabilitative Care (QuIRC): a measure of best practice for facilities for people with longer term mental health problems

**DOI:** 10.1186/1471-244X-11-35

**Published:** 2011-03-01

**Authors:** Helen Killaspy, Sarah White, Christine Wright, Tatiana L Taylor, Penny Turton, Matthias Schützwohl, Mirjam Schuster, Jorge A Cervilla, Paulette Brangier, Jiri Raboch, Lucie Kališová, Georgi Onchev, Spiridon Alexiev, Roberto Mezzina, Pina Ridente, Durk Wiersma, Ellen Visser, Andrzej Kiejna, Tomasz Adamowski, Dimitri Ploumpidis, Fragiskos Gonidakis, José Caldas-de-Almeida, Graça Cardoso, Michael B King

**Affiliations:** 1 Research Department of Mental Health Sciences, UCL Medical School, London, UK; 2 Division of Mental Health, St. George's University London, London, UK; 3 Department of Psychiatry and Psychotherapy, University Hospital Carl Gustav Carus, Technische Universitaet Dresden, Dresden, Germany; 4 Mental Health Unit, San Cecilio University Hospital, University of Granada, Spain; 5 CIBERSAM, Universidad de Granada, Granada, Spain; 6 Psychiatric Department of the First Faculty of Medicine, Charles University, Prague, Czech Republic; 7 Department of Psychiatry, Medical University Sofia, Sofia, Bulgaria; 8 Dipartimento di Salute Mentale, University of Trieste, Trieste, Italy; 9 Psychiatry, University Medical Centre Groningen, University of Groningen, Groningen, Netherlands; 10 Department of Psychiatry, Wroclaw Medical University, Wroclaw, Poland; 11 University Mental Health Research Institute (UMHRI), Athens, Greece; 12 Department of Mental Health, Faculdade de Ciencias Medicas, New University of Lisbon, Lisbon, Portugal

## Abstract

**Background:**

Despite the progress over recent decades in developing community mental health services internationally, many people still receive treatment and care in institutional settings. Those most likely to reside longest in these facilities have the most complex mental health problems and are at most risk of potential abuses of care and exploitation. This study aimed to develop an international, standardised toolkit to assess the quality of care in longer term hospital and community based mental health units, including the degree to which human rights, social inclusion and autonomy are promoted.

**Method:**

The domains of care included in the toolkit were identified from a systematic literature review, international expert Delphi exercise, and review of care standards in ten European countries. The draft toolkit comprised 154 questions for unit managers. Inter-rater reliability was tested in 202 units across ten countries at different stages of deinstitutionalisation and development of community mental health services. Exploratory factor analysis was used to corroborate the allocation of items to domains. Feedback from those using the toolkit was collected about its usefulness and ease of completion.

**Results:**

The toolkit had excellent inter-rater reliability and few items with narrow spread of response. Unit managers found the content highly relevant and were able to complete it in around 90 minutes. Minimal refinement was required and the final version comprised 145 questions assessing seven domains of care.

**Conclusions:**

Triangulation of qualitative and quantitative evidence directed the development of a robust and comprehensive international quality assessment toolkit for units in highly variable socioeconomic and political contexts.

## Background

Worldwide, countries are at different stages of deinstitutionalisation [1] and in Europe, despite the investment in community services, many individuals with mental health problems still live in asylums or other types of institutions [2]. The majority have longer term conditions [3] with complications such as treatment resistance [4], cognitive impairment and pervasive negative symptoms [5], poor function [6], substance misuse and challenging behaviours [7]. They are at risk of abuse of their human rights since their capacity to make informed choices about their care may be impaired. The European Commission's Green Paper [8] on improving the mental health of the population highlighted the importance of promotion of social inclusion of the mentally unwell and protection of their rights and dignity. This paper reports on the development of an international toolkit to assess the quality of care delivered in hospital and community based mental health units.

## Methods

The Development of a European Measure of Best Practice for people with longer term mental health problems in institutional care (DEMoBinc) was a three year project funded by the European Commission from March 2007. It involved eleven centres across ten countries at different stages of deinstitutionalisation (Bulgaria, Czech Republic, Germany, Greece, Italy, Netherlands, Poland, Portugal, Spain, UK). Full details of the study protocol are published elsewhere [9]. In summary, the project comprised six phases: 1) identification of the domains of care for inclusion in the toolkit through triangulation of the results of i) a review of care standards in each country, ii) a systematic literature review of the components of care (and their effectiveness) in mental health institutions, and iii) a Delphi exercise with four stakeholder groups in each country (service users, carers, professionals, advocates) on the aspects of care that promote recovery for people with mental health problems living in institutions; 2) piloting and testing the inter-rater reliability of the toolkit; 3) refining the toolkit; 4) testing the association between toolkit ratings (gathered from the facility's manager) with service users' experiences of care, quality of life, autonomy and markers of recovery; 5) assessing the toolkit's ability to report on a facility's "value for money" through a health economic analysis; 6) dissemination of results. This paper reports on the first three phases.

### Phase 1

The results of the systematic review of the literature on components of institutional care have been published elsewhere [10]. Eight domains of care were identified: living conditions; interventions for schizophrenia; physical health; restraint and seclusion; staff training and support; therapeutic relationship; autonomy and service user involvement; and clinical governance. The results of the Delphi exercise have also been previously reported [11] and eleven domains of care were identified: social policy and human rights; social inclusion; self management and autonomy; therapeutic interventions; governance; staffing; staff attitudes; therapeutic environment; post-discharge care; carers; physical health care [11]. Collation of each country's care standards by HK and TT identified seven domains: living environment; mental and physical health; therapeutic relationship; service users' rights and autonomy; service user involvement; staff training and support; clinical governance. The project steering committee (PSC) reviewed these findings and agreed on nine domains for inclusion in the toolkit (Living Environment; Treatments and Interventions including restraint and seclusion; Therapeutic Environment; Self-management and Autonomy; Social Policy, Citizenship and Advocacy; Clinical Governance; Social Interface; Human Rights; and Recovery Based Practice). These were further reviewed and agreed by an international panel of experts in social care, mental health rehabilitation, recovery based practice, service user experience, disability rights, international mental health law, international mental health policy and care standard setting.

Toolkit items for assessment of these domains were generated by the UK centres. The toolkit was designed to be completed by the manager of the facility since we were aware, due to the complexity of their mental health problems, that only some service users would have the capacity to complete such a measure. However, service users' experiences of care were assessed in a later Phase of the project to investigate the association between unit manager toolkit ratings and service user reports. Where possible, toolkit items were worded to avoid revealing which answer would lead to a higher quality rating. A mix of question formats was used (Likert scales, ordered categories, quantitative responses, binary responses, lists of yes/no's summed to create quantitative responses, and vignettes that asked the respondent to generate answers which were "checklisted" by the researcher and summed to give a quantitative response). The varied format of questions aimed to increase the accuracy of responses by avoiding a response set and make the toolkit more interesting to complete. The draft toolkit was reviewed by the PSC and the international expert panel and further questions were added if there was evidence for their inclusion from Phase 1 or if they appeared highly relevant across countries.

The toolkit was translated in each country and back translated by someone independent of the project. Back translations were reviewed at the lead centre in the UK and amendments agreed with each country. The toolkit was piloted in each country in one or two facilities. A training session was attended by all researchers involved in data collection to ensure clarity of understanding of all items and their scoring.

### Phase 2

The draft toolkit comprised 154 questions (consisting of 280 items) of which 29 were descriptive and did not contribute to scoring. The remaining questions were allocated to one or more of the nine domains by the UK research teams. Since some questions were combined for the purposes of scoring, a total of 96 question scores contributed to the rating of domains. Of these, 27 assessed only one domain, 32 assessed two domains, 18 assessed three, 17 assessed four and two assessed five. Since the toolkit had a variety of response structures, questions were scored within a similar range to ensure similar weighting of items within each domain. For example, Likert scale responses were transformed from a scale of 1 to 5 to -2 to +2.

Each country identified 20 facilities (units) in which to carry out inter-rater reliability testing of the draft toolkit that: provided for adults with longer term mental health problems (length of stay at least six months); had at least six patients/residents; had communal facilities; had staff on site, ideally 24 hours per day. Units that only provided for specialist groups (e.g. learning disability or dementia) were excluded. Hospital and community based units were recruited to give a range in size and geographical spread within countries. Sampling was not random; units were identified from registration lists in each country and/or were known to the lead investigator in each country.

Face to face interviews to complete the draft toolkit were carried out by the researchers with the manager of each unit. Inter-rater reliability was tested in one of three ways; a second researcher was also present at the interview and completed ratings simultaneously, or they repeated the interview with the manager within two weeks, or they rated the toolkit from a tape recording of the first interview. Researchers were not allowed to confer on ratings of the same unit. Feedback from interviewees and researchers was collected on the relevance and usefulness of the toolkit questions, the ease of completion and the time taken to complete.

#### Data management and analysis

A common SPSS database was developed in the lead centre and distributed to all centres. A test entry of pilot data in each centre clarified any coding queries. Double data entry was completed for 10% of the toolkit data using a separate database and the study statistician carried out data validation on the two databases for each centre. The maximum error rate was set at 5%. Any centre that had an error rate above this was required to complete double data entry for all their data.

Inter-rater reliability of toolkit items was assessed using the Kappa coefficient for categorical data (weighted Kappa where there were more than two categories) and the intraclass correlation coefficient (ICC) for normally distributed, continuous data. Paired ratings for 20 institutions in 10 countries (200 institutions in all) enabled a 95% confidence interval for the estimate of ICC of ± 0.15 [12]. Items whose Kappa was below 0.4 or ICC/weighted Kappa was below 0.7 were dropped. Items that had a narrow spread (categorical items with more than 90% of the response or Likert scale items where >80% of responses fell to either side of neutral) were also dropped due to their inability to discern differences in quality between units.

The fact that many questions contributed to the rating of more than one domain meant domains were likely to be highly correlated with each other rather than assessing discrete aspects of care. An exploratory factor analysis (EFA) was therefore indicated to explore the latent factor structure of the 96 scored questions, reduce the overlap between domain content and ensure common variation of items within a domain. However, using the five subjects per item rule of thumb for EFA, a sample size of at least 500 units would have been required. An iterative EFA was therefore carried out which could take account of the available sample size.

The first iteration of the EFA used a Principal Components Analysis of each domain, extracting factors indicated by Velicers MAP [13]. No rotation was necessary as there was no intention to interpret the factors extracted. Having completed this for each domain, the unrotated factor loadings were examined. A factor loading greater than 0.3 was taken to indicate that the item was correlated with other items in the domain. Since many items were initially allocated to more than one domain, our first approach to reducing the overlap between domains was to identify items which did not load onto their allocated domain. Such items were removed from that domain as long as they loaded onto another domain. Items which did not load onto any domain in the first iteration could potentially load onto their allocated domains once other items had been removed. The procedure was therefore repeated and an assessment of factor loadings from this second iteration was conducted as before and items that did not load were removed. The third and final iteration was carried out as before but this time all items with a factor loading less than 0.3 were removed even if this meant that they were not retained in any domain. Based on this third iteration a final allocation of items to domains was produced. The reliability of these domains was assessed using two measures: 1) the KMO measure of sampling adequacy and 2) Cronbach's Alpha, a measure of internal consistency. A value of greater than 0.7 is desirable for both.

### Phase 3

The toolkit was refined in light of a) the feedback from interviewers and unit managers b) the results of the inter-rater reliability testing c) the results of the EFA. Amendments were discussed and agreed by the PSC and international expert panel.

## Results

In total, 202 units were recruited across the ten countries. No centre had a data entry error rate over 5% and no complete double data entry was required. Of the 202 units, 93 (46%) were in the inner city, 73 (36%) in the suburbs and 37 (18%) in the country. The majority (120, 59%) were community based, 47 (23%) were hospital wards and 35 (17%) were units within the hospital grounds. Their size ranged from five to 320 beds (mean 30, median 19); 162 (80%) had no maximum length of stay and of those that did the mean was 1.8 years (range 0.5 to 5, median 2). Thirty-three (16%) units were for men only and 18 (9%) for women only. Table 1 shows the characteristics of units recruited in each country. Independent data collection for inter-rater reliability testing of the toolkit was carried out in only one case by a second rater repeating the interview.

**Table 1:** Characteristics of included units and inter-rater reliability testing method

Country	Units approached	Units recruited	Hospital units recruited	Community units recruited	Houses/units on hospital grounds recruited	Number of units where both researchers were present at interview	Number of units where second researcher coded a recorded interview
**UK**	24	20	2 (10%)	13 (65%)	5 (25%)	16 (80%)	4 (20%)

**Germany**	26	20	0	19 (1%)	1 (5%)	0	20 (100%)

**Spain**	20	20	4 (20%)	11 (55%)	5 (25%)	20 (100%)	0

**Czech Republic**	21	21	15 (71%)	6 (29%)	0	8 (38%)	13 (62%)

**Bulgaria**	21	20	8 (40%)	10 (50%)	2 (10%)	0	19* (95%)

**Italy**	20	20	0	15 (75%)	5 (25%)	12 (60%)	8 (40%)

**Netherlands**	22	21	0	12 (57%)	9 (43%)	6 (29%)	15 (32%)

**Poland**	26	20	17 (85%)	3 (15%)	0	2 (10%)	18 (90%)

**Greece**	22	20	0	20 (100%)	0	20 (100%)	0

**Portugal**	20	20	1 (5%)	11 (55%)	8 (40%)	5 (25%)	15 (75%)

**Total**	**222**	**202**	**47 (23%)**	**120 (59%)**	**35 (17%)**	**89 (44%)**	**112 (55%)**

* In only 1 unit (in Bulgaria) toolkit inter-rater reliability was assessed by two researchers interviewing the unit manager separately

**Figure 1:**
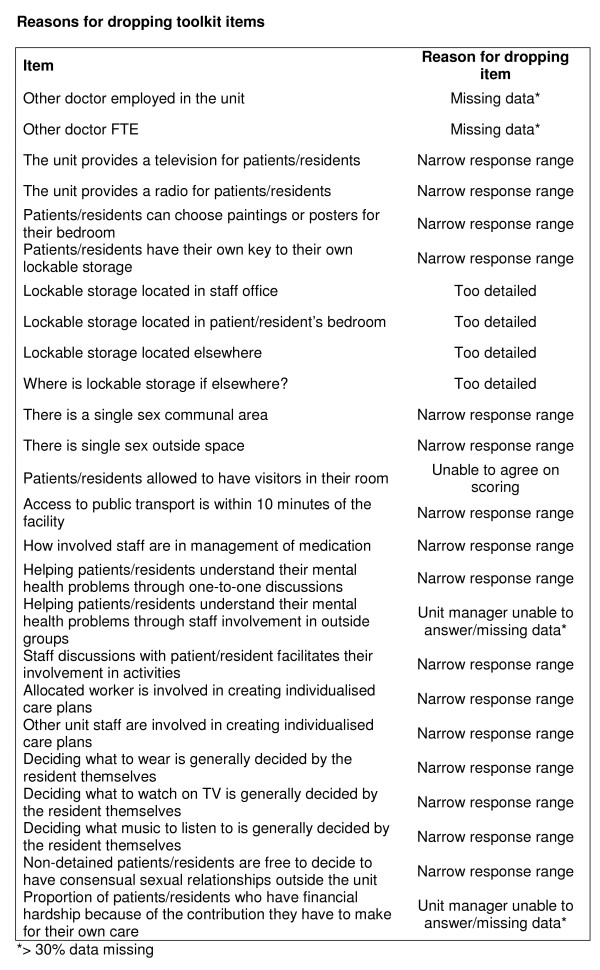
**Reasons for dropping toolkit items**.

Sixteen items had a narrow range of response (Figure 1).

The results of the inter-rater reliability testing are shown in Additional file 1. Only one item had poor inter-rater reliability (How many CBT appointments are usually offered?) but was retained with an amended response structure.

Of the 202 managers interviewed, 189 (94%) thought the toolkit questions were relevant/very relevant to their unit and 178 (88%) thought the results would be useful/very useful in auditing the quality of their unit. Of the 202 interviews carried out, the researchers reported that 143 (71%) took between one and two hours, 43 (21%) took less than an hour and 15 (7%) took over two hours. There were problems in accessing information in 37 (18%) interviews.

The toolkit was refined through discussion with the PSC and international expert panel in light of the results. The 16 items with a narrow range of response were dropped and nine others were dropped for the reasons shown in Figure 1. Eight items were merged with another item, three items were amended from single answer to categorical response options and one item was added (total number of staff employed by or visiting the unit). The final toolkit comprised 145 questions.

In the initial allocation of scored items to domains, 25 were allocated to Living Environment, 42 to Therapeutic Environment, 34 to Treatments and Interventions, 32 to Self-management and Autonomy, eight to Social Policy and Citizenship, eight to Clinical Governance, 19 to Social Interface, 30 to Human Rights and 25 to Recovery Based Practice. The following pairs of domains shared more than 50% of items: all Social Policy, Citizenship and Advocacy questions were also in Human Rights; 72% of Recovery Based Practice questions were in Therapeutic Environment; 64% of Recovery Based Practice questions were in Self-management and Autonomy; 60% of Human Rights questions were in Self-management and Autonomy; 53% of Social Interface questions were in Treatments and Interventions; 50% of Clinical Governance questions were in Human Rights and 50% were in Therapeutic Environment.

**Figure 2:**
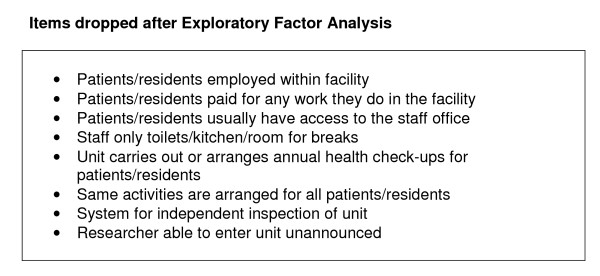
**Items dropped after Exploratory Factor Analysis**.

After the first iteration of the EFA, 16 items were removed from domains they did not load onto where they loaded onto another domain. After the second iteration one item (is there a private room for patients/residents to meet with their visitors?) which had not loaded onto any domain in the first iteration now loaded onto Living Environment and was retained. One question (unit has a policy for dealing with a report from a patient/resident of abuse, aggression or bullying from a member of staff?) which had loaded onto Clinical Governance and Human Rights after the first iteration now did not load onto Clinical Governance and was retained only in Human Rights. One item (unit provides the same activities for all residents?) which had loaded onto Therapeutic Environment after the first iteration no longer loaded after the second iteration. Eight items which did not load onto any domain after the first and second iterations were dropped (Figure 2) and the third iteration of EFA run. This indicated that all remaining items loaded onto at least one domain with a factor loading greater than 0.3.

The KMO measures of sampling adequacy of the nine domains were low for Clinical Governance and Social Policy, Citizenship and Advocacy (0.52 and 0.61 respectively). Clinical Governance comprised only three items and Social Policy, Citizenship and Advocacy comprised six. All these items also contributed to other domains. The PSC therefore agreed that these two domains could be dropped without the loss of any toolkit content. The KMO statistics for the remaining seven domains ranged from 0.67 to 0.80 with only one (Social Interface) falling just below 0.7. The number of items per domain, KMO and Cronbach's Alpha statistics are shown in Table 2. These demonstrate that all seven domains had good internal consistency (again only Social Interface fell just below the threshold of 0.7). The final allocation of questions to domains comprised 88 questions allocated to one or more of seven domains (38 were allocated to one domain, 24 to 2, 20 to 3, 5 to 4 and 1 to 5). The EFA process reduced the overlap of items between domains (57% of Recovery Based Practice items in Self-management and Autonomy compared with 64% originally; 52% of Human Rights in Self-management and Autonomy compared with 60% originally; 71% of Recovery Based Practice items in Therapeutic Environment compared with 72% originally; 60% of Social Interface items in Treatments and Interventions compared with 53% originally).

**Table 2:** Sampling adequacy and internal consistency of domains after 3^rd ^iteration of exploratory factor analysis

Domain	Number of items	KMO statistic	Cronbach's alpha
**Living Environment**	22	0.77	0.82

**Therapeutic Environment**	36	0.70	0.76

**Treatments and Interventions**	28	0.74	0.70

**Self-management and Autonomy**	28	0.80	0.86

**Social Interface**	10	0.67	0.65

**Human Rights**	24	0.74	0.78

**Recovery Based Practice**	20	0.72	0.77

## Discussion

The project facilitated the development of the first international quality assessment toolkit for longer term hospital and community based mental health facilities, the Quality Indicator for Rehabilitative Care (QuIRC). The toolkit has excellent inter-rater reliability and since items were derived from the results of a systematic literature review, Delphi exercises with stakeholder groups in a diverse range of countries, and a review of care standards in each country, the toolkit is able to deliver comprehensive assessment of units in countries at different stages of deinstitutionalisation.

The exploratory factor analysis provided a data driven corroboration and refinement of our original allocation of items to domains and reduced the overlap of content between domains. Although overlap of items in sub-scores of assessment tools is not usual, we feel it is acceptable for specific aspects of care to contribute to the quality rating of more than one domain since this reflects the multiple effects of the complex interventions delivered in facilities for those with more complex mental health problems. Three domains shared the greatest content with other domains (Social Interface, Human Rights and Recovery Based Practice) which highlights their "cross-cutting" nature.

The total QuIRC score provides a measure of overall quality of care and domain scores indicate where specific improvements may be required. A web based version of the QuIRC is available in ten languages that compares the unit's domain scores with similar units in the same country (http://www.quirc.eu). This allows its use as a local, regional and national quality assessment tool and it has been incorporated into the UK's peer accreditation process for inpatient mental health rehabilitation units. It is also being used in a national programme of research of these units in England.

## Conclusions

Triangulation of qualitative and quantitative evidence directed the development of a robust and comprehensive international quality assessment toolkit for facilities providing care for people with longer term mental health problems in highly variable socioeconomic and political contexts. The QuIRC represents the first measure of this type and has potential for use as a research tool and as an international quality benchmark.

## Competing interests

The authors declare that they have no competing interests.

## Authors' contributions

HK, MK, CW and SW conceived and designed the study. SW carried out the data analysis. HK drafted the article which was reviewed and revised by all authors. All authors agreed the final version for publication.

## Pre-publication history

The pre-publication history for this paper can be accessed here:

http://www.biomedcentral.com/1471-244X/11/35/prepub

## Supplementary Material

Additional file 1 **Results of inter-rater reliability testing**. Click here for file
